# Glutaric Acidemia, Pathogenesis and Nutritional Therapy

**DOI:** 10.3389/fnut.2021.704984

**Published:** 2021-12-15

**Authors:** Qian Li, Chunlan Yang, Lijuan Feng, Yazi Zhao, Yong Su, Hong Liu, Hongkang Men, Yan Huang, Heinrich Körner, Xinming Wang

**Affiliations:** ^1^Department of Pharmacy, Suizhou Hospital, Hubei University of Medicine, Suizhou, China; ^2^Department of Pharmacy, First Affiliated Hospital of Anhui Medical University, Hefei, China; ^3^School of Pharmacy, Anhui Medical University, Hefei, China; ^4^Key Laboratory of Anti-inflammatory and Immune Medicine, Anhui Collaborative Innovation Center of Anti-inflammatory and Immune Medicine, Institute of Clinical Pharmacology, Ministry of Education, Anhui Medical University, Hefei, China

**Keywords:** Glutaric acidemia, pathogenesis, genetic disorders, maintenance therapy, nutrition therapy

## Abstract

Glutaric acidemia (GA) are heterogeneous, genetic diseases that present with specific catabolic deficiencies of amino acid or fatty acid metabolism. The disorders can be divided into type I and type II by the occurrence of different types of recessive mutations of autosomal, metabolically important genes. Patients of glutaric acidemia type I (GA-I) if not diagnosed very early in infanthood, experience irreversible neurological injury during an encephalopathic crisis in childhood. If diagnosed early the disorder can be treated successfully with a combined metabolic treatment course that includes early catabolic emergency treatment and long-term maintenance nutrition therapy. Glutaric acidemia type II (GA- II) patients can present clinically with hepatomegaly, non-ketotic hypoglycemia, metabolic acidosis, hypotonia, and in neonatal onset cardiomyopathy. Furthermore, it features adult-onset muscle-related symptoms, including weakness, fatigue, and myalgia. An early diagnosis is crucial, as both types can be managed by simple nutraceutical supplementation. This review discusses the pathogenesis of GA and its nutritional management practices, and aims to promote understanding and management of GA. We will provide a detailed summary of current clinical management strategies of the glutaric academia disorders and highlight issues of nutrition therapy principles in emergency settings and outline some specific cases.

## Introduction

Both types of Glutaric acidemia (GA) are autosomal recessive, metabolic disorders. Glutaric acidemia type I (GA-I) is a katabolic deficiency of the L-lysine, L-hydroxylysine and L-tryptophan metabolism that was first described in 1975 (1). Its worldwide incidence is ~1 in 110,000, which makes it one of the more common inherited metabolic disorders that in total affect one out of 30,000–100,000 children (2). GA- I results primarily, in the accumulation of glutaric acid (GA) and 3-hydroxyglutaric acid (3-OH-GA) in the urine, and secondarily, in carnitine deficiency (3). Children affected by this disorder, may experience an encephalopathic crisis that follows a period of seemingly normal development and can result in an irreversible striatal injury (2). The second form, Glutaric acidemia type II (GA-II), also known as Multiple Acyl-CoA Dehydrogenase Deficiency (MADD), was first described in 1976 in an infant with nonketotic hypoglycemia, metabolic acidosis, and strong “sweat-sock” odor (4). GA-II is an inherited deficiency of acyl-CoA dehydrogenases, such as short-, medium-, and long-chain acyl CoA dehydrogenases, which is caused by a defect in either electron transfer flavoprotein (ETF) or electron transfer flavoprotein dehydrogenase (ETFDH) (5). This defect results in compromised fatty acid, amino acid, and choline metabolism with consequent impaired adenosine triphosphate (ATP) synthesis, insufficient gluconeogenesis and excessive lipid accumulation in different organs (6). Advances in both nutritional therapy and disease management have considerably improved the clinical outcome of GA. This article reviews the progress in our understanding of the pathogenesis of GA (type I and II), respectively, and how it can be countered by management practice, both in everyday care and emergency situations.

## GA-I

### Pathogenesis of GA-I

GA-I presents as a severe neurometabolic aciduria which is characterized by acute encephalopathic crises in early childhood. This disease is caused by an inherited deficiency of the flavoprotein Glutaryl-CoA dehydrogenase (GCDH; EC 1.3.99.7) (7). Mature GCDH exists in the mitochondrial matrix as a homo-tetramer of 43.3-kDa subunits, each of which is derived from a 48.2-kDa precursor peptide by cleavage of a 44-amino acid mitochondrial targeting sequence from its N-terminus (8). The enzyme is encoded by the GCDH gene that is located at the gene locus 19p13.2. The gene stretches over 7 kb comprising 11 exons and 10 introns (9).

**Figure 1:**
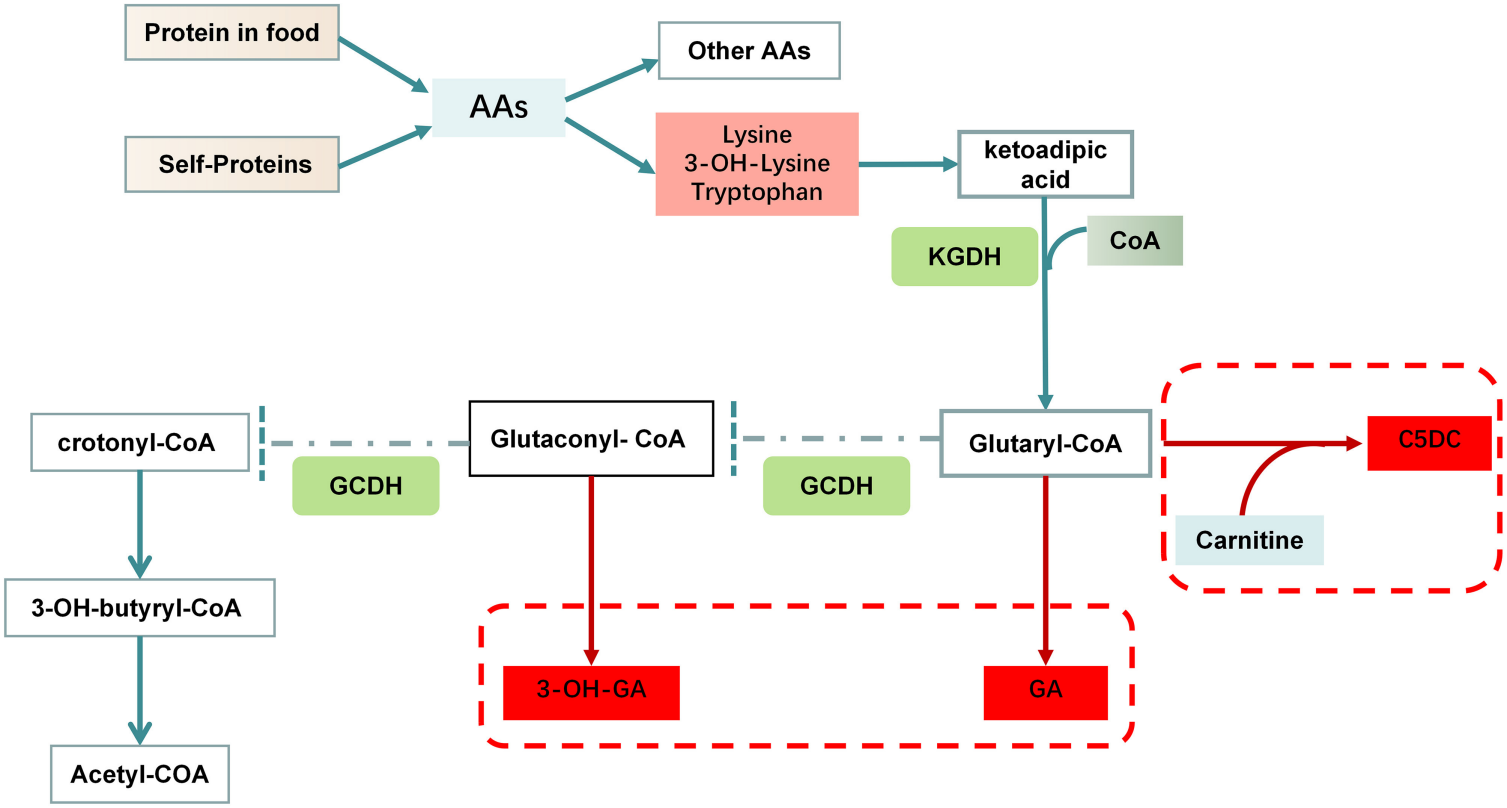
Disorders of lysine and tryptophan metabolism in GA-I. Lysine and tryptophan enter cells through distinct sodium-independent facilitative amino acid transporters. These amino acids are converted to ketoadipic acid (KA) in the cytosol through glutamate/ketoglutarate-coupled transamination and transported into mitochondria. Subsequently, KA is oxidatively decarboxylated under the catalysis of α-ketoglutarate dehydrogenase (KGDH). It utilizes free CoA to form glutaryl-CoA, which can undergo conjugation with carnitine to form C5DC. Glutaryl-CoA dehydrogenase catalyzes the conversion of glutaryl-CoA to crotonyl-CoA in a two-step reaction, first dehydrogenated to glutaconyl-CoA, and second decarboxylated to crotonyl-CoA. A mutated GCDH enzyme cannot metabolize glutaryl-CoA to crotonyl-CoA resulting in accumulation of GA, glutaryl-CoA and 3-OH-GA, which have been proposed to act as endogenous neurotoxins.

GCDH is a multifunctional enzyme that catalyzes both the oxidation of glutaryl-CoA to glutaconyl-CoA and the subsequent decarboxylation of the latter compound to crotonyl-CoA in the degradative pathway of L-lysine, L-hydroxylysine and L-tryptophan metabolism (7, 10) (Figure 1). GCDH deficiency was not an isolated defect of the decarboxylation step, but either an isolated defect of oxidation or a combined defect of both the oxidation and decarboxylation steps (11). Deficient GCDH activity results in an accumulation of GA, 3-OH-GA and a lesser extent glutaconic acid and glutaryl carnitine (C5DC) in body fluids and brain (3). Studies have demonstrated that accumulation of GA, 3-OH-GA and glutaryl-CoA interferes with cerebral energy metabolism (12). Because GA and 3-OH-GA are only weak neurotoxins the neurodegenerative cascade destroying the striatum in patients with GA-I involves mainly mechanisms other than excitotoxicity (13). Data from 215 patients diagnosed with GA-I showed that good correlations between genotype and biochemical phenotype, GA and 3-OH-GA concentrations in plasma and urine negatively correlate with residual GCDH activity in fibroblasts and leucocytes. There was no clear correlation with the severity of clinical symptoms (14). Furthermore, the severity of the clinical phenotype usually depends on the development of an encephalopathic crisis in childhood. The early onset of gradual motor symptoms (6–9 months) and brain atrophy are poor prognostic signs, the age at symptom onset can significantly predict the severity of motor deficits and the overall outcome (15). A depletion of free carnitine occurs as a consequence of the formation of C5DC (Figure 1).

### Nutrition Therapy

During the last three decades, therapeutic goals have been established and optimized, which have been recommended by an international guideline group (16). Cerebral accumulation of GA and 3-OH-GA is considered as a biochemical risk factor for the manifestation of neurodegeneration in GCDH deficiency and therapeutic strategies to decrease these metabolites will be neuroprotective. The combined metabolic treatment of GA-I includes emergency treatment and long-term maintenance therapy. Early emergency treatment commenced during threatening episodes has the aim to prevent or quickly reverse a catabolic crisis, usually induced by infectious diseases, surgery, or an inflammatory response to vaccinations. This treatment is followed by maintenance therapy that combines low lysine diet with lysine-free, tryptophan-reduced amino acid supplements (AAS) and prevention of secondary carnitine depletion by carnitine supplementation. This regime of intensive emergency treatment combined with subsequent L-carnitine supplementation during the course of infectious diseases has decreased the frequency of acute encephalopathic crises from 90–95% in untreated to 5–35% in early diagnosed and appropriately treated patients (17). The treatment strategies in conditions of the GA-I are summarized in Table 1.

**Table 1:** The currently treatment strategies of GA-I.

**GA-I**	**Maintenance treatment**						**Emergency treatment**
Age	Child					Adult	0–6 years
	0–6 month	7–12 months	1–3 years	4–6 years	>6 years		
Diet	low-lysine diet						
Lysine (from food)	100 (mg/kg/d)	90 (mg/kg/d)	60–80 (mg/kg/d)	60–50 (mg/kg/d)	Low lysine diet while ensuring adequate essential nutrients to meet the growing needs	30 (mg/kg/d)	Stop diet of 24–48 h, iv glucose to reverse catabolism (iv 0.025–0.05 IU insulin/kg/h if persistent hyperglycemia and/or glucosuria).
Special amino acid mixtures (AAS)	0.8–1.3 (g/kg/d)	0.8–1.0 (g/kg/d)	0.8 (g/kg/d)	0.8 (g/kg/d)		—	See maintenance treatment
Carnitine	100 (mg/kg/d)	100 (mg/kg/d)	100 (mg/kg/d)	100–50 (mg/kg/d)	50–30 (mg/kg/d)	30 (mg/kg/d)	100 (mg/kg/d. iv)
Arginine	No reliable evidence					300–600 (mg/kg/d)	—

#### Maintenance Therapy

When GA-I is suspected during diagnosis, e.g., increased concentration of 3-OH-GA in urine, metabolic treatment should be started immediately, 80–90% of individuals with GA-I remain asymptomatic if treatment has started before onset of symptoms in newborns, but when a diagnosis is made after neurologic symptoms have appeared, therapeutic impact is usually limited and outcome will be poor. Individuals adhering to treatment recommendations rarely develop dystonia (5%), while non adherence to maintenance treatment increases the rate to 44% and nonadherence to emergency treatment to 100% (18). Most crises occur from age 3 months to 3 years, with only a small percentage being reported between an age from 3 to 6 years and no crisis being reported beyond the age of 6 years (2). Dietary treatment recommendations are considering age-dependent needs of a growing child.

##### Age Over 6 Years

*Dietary Treatment*. Low lysine diet is used to reduce neurotoxic GA and 3-OH-GA, while ensuring adequate intake of essential nutrients and energy substrates that meet the needs of a growing child. Concurrent limitation of protein consumption reduces lysine intake further. Since the lysine content in natural foods varies considerably, e.g., 2–4% (lysine/protein) in cereals and 9% (lysine/protein) in fish, a direct calculation of lysine intake instead of total natural protein intake is more precise (19). Tryptophan content in natural protein is only 0.6–2%; its quantification in plasma is technically challenging, and depletion may cause severe neurologic deficits (20). Therefore, dietary amino acid mixtures (AAM) used for treatment should be tryptophan reduced but not tryptophan free. Therefore, individuals with GA-I receive lysine-free, tryptophan-reduced AAM which aim to provide adequate supply of essential amino acids. In a current study, the intake of lysine-low diet in combination with a lysine-free AAM recommended for GA-I is 100 mg/kg/d of lysine, 1.3–0.8 g/kg/d AAM for 0–6 months; 90 mg/kg/d of lysine, 1.0-0.8g/kg/d AAM for 7–12 months; 60–80 mg/kg/d of lysine, 0.8 g/kg/d AAM for 1–3 years; 50–60 mg/kg/d of lysine, 0.8 g/kg/d AAM for 4–6 year of age (16) (Table 1). A Lysine-low diet in combination with lysine-free, tryptophan-reduced AAS prevents malnutrition and promotes normal gain of weight in asymptomatic children whereas linear growth might be slightly compromised in some of them. In contrast, individual nutritional concepts are required for dystonic patients to prevent inappropriate weight gain and poor growth (19).

**Figure 2:**
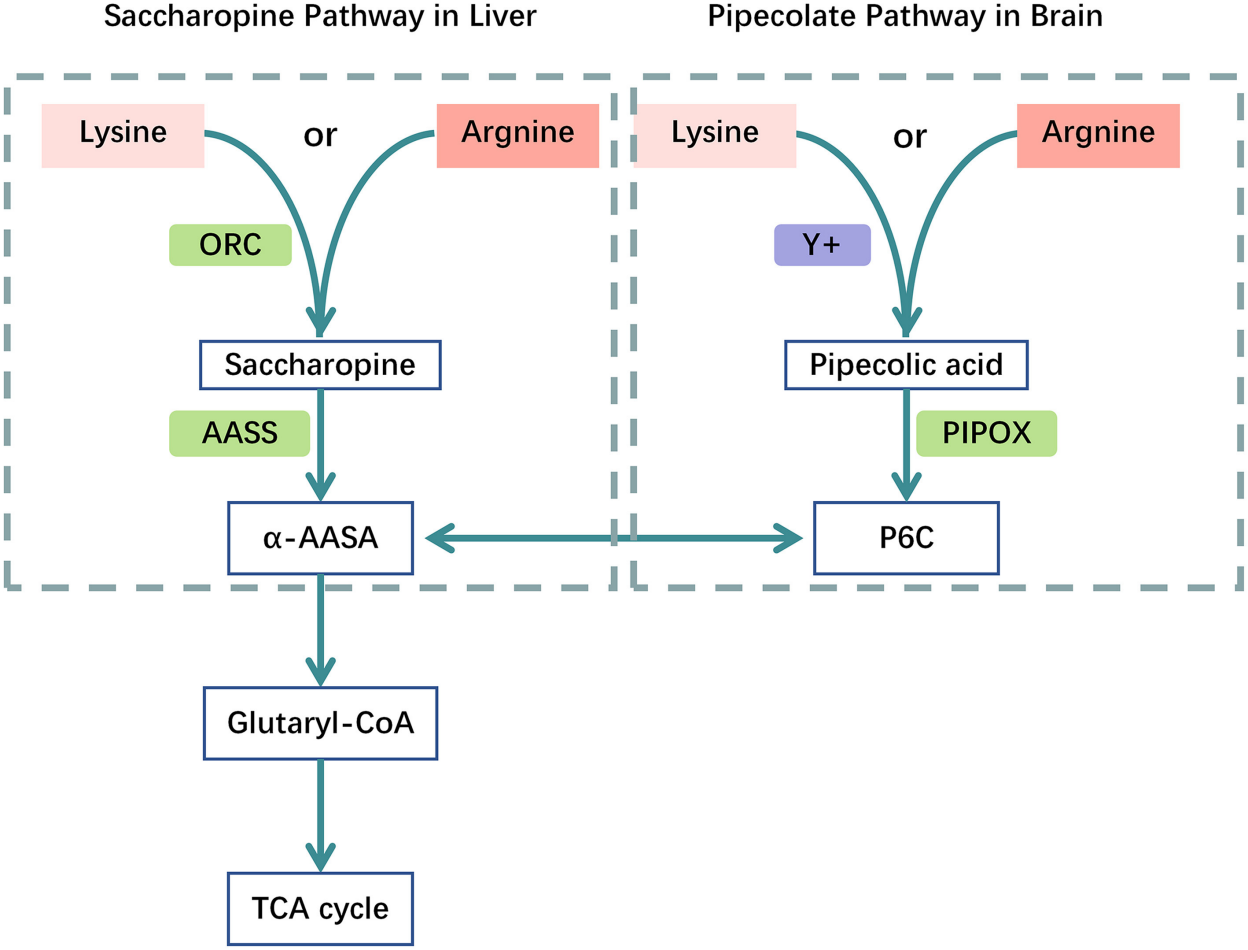
Catabolic pathways of the Lysine metabolism. Competition between L-arginine and L-lysine at the mitochondrial membrane and the blood-brain barrier. Catabolic pathways: (1) the mitochondrial saccharopine pathway is the major route in liver; (2) the peroxisomal pipecolate pathway is the major route in brain, both branches converge in α-AASA and its cyclic equivalent P6C. Acetyl-CoA is the ultimate product of the lysine degradation. human mitochondrial ornithine carriers 1 and 2 (ORC); system y+ of the blood–brain barrier (y+), pipecolate oxidase (PIPOX), AASS, α-aminoadipate semialdehyde synthase (AASS); piperideine-6-carboxylate (P6C);α-aminoadipate semialdehyde (α-AASA); α-AAA, α-aminoadipate (α-AAA); tricarboxylic acid-cycle (TCA cycle).

*Arginine*. In humans, lysine is catabolized *via* two separate pathways; the saccharopine pathway mainly located in the liver, and the pipecolic acid pathway mainly located in the brain. Both pathways converge at the level of α-aminoadipic acid (21, 22). L-lysine and L-arginine compete for system y+ localized in the blood-brain barrier to enter the brain or for mitochondrial L-ornithine carriers 1 (and 2) at the mitochondrial membrane in liver, respectively (Figure 2) (21–23). In a mouse model, low L-lysine diet decreased the concentrations of GA in and brain in a dose-dependent manner, whereas 3-OH-GA concentrations remained virtually unchanged, L-arginine supplementation amplified this biochemical effect and thus further decreased the cerebral and hepatic GA concentrations and 3-OH-GA concentration (23). A cohort study incorporating 168 GA-I patients over 31 years indicated that nutritional management with lysine-free, arginine-enriched metabolic formula and emergency IV infusions during the first 2 years of life is safe and effective, preventing more than 90% of striatal injuries while supporting normal growth and psychomotor development. Based on the authors calculations, dietary management with Lys–Arg+ formula could decrease the brain's toxin exposure by as much as 40% during its vulnerable phase of development (24). Adequate doses of arginine required to competitively inhibit enteral lysine uptake is not clear, in healthy humans, may need 300–600 mg/kg/d of L-arginine HCl and lysine intake restricted to DRI (30 mg/kg/d) to reduce enteral lysine uptake and systemic lysine oxidation (21). There is no reliable evidence to support the recommended dose for arginine in GA-I yet.

*L- Carnitine*. L- carnitine supplementation alone even at high doses is insufficient to lower GA and 3-OH-GA levels (23). Carnitine supplementation combined with glutaryl-CoA is considered a physiological detoxification to form nontoxic C5DC, meanwhile to prevents secondary depletion of free carnitine and improves the outcome (25). The current recommendation for carnitine intake in the treatment of GA-I is 100mg/kg/d for <4 years, 50–100mg/kg/d for 4–6 years, 30–50 mg/kg/d for >6 year of age (16) (Table 1). A 4.5-year-old girl who received L-carnitine (50 mg/kg), protein/amino acid supplement (60 g/d) and was on a predominantly vegetarian and protein diet was admitted to hospital for recurrent rhabdomyolysis three times within 3 years. After the third admission after diagnosis of GA-1, this child had normal growth and development without symptoms during a 3.5 year of follow-up. This extremely rare rhabdomyolysis case in combination with GA-I had neither obvious manifestations of the condition nor any adverse neurological outcomes (26).

##### Age Over 6 Years

The long-term outcome in GA-I is largely unknown. Acute and insidious disease onset manifest during the first 6 years of life, whereas individuals with late onset often present during adolescence or adulthood. After age 6 years, dietary treatment should follow the principle of an age-adapted, protein-controlled (natural protein with a low lysine content and avoiding lysine-rich food) program to prevent growth disturbance or malnutrition (16).

#### Emergency Therapy

Irreversible neurological symptoms generally occur acutely between age 3 months and 3 years during encephalopathic crises and are usually caused by events that may induce a catabolic state (e.g., febrile illness in particular gastroenteritis, febrile reactions to vaccination, or perioperative/peri-interventional fasting periods) (3, 16), emergency treatment should be initiated without delay after the onset of first symptoms of acute intercurrent illness. these crises result in irreversible striatal injury and, subsequently, a complex, movement disorder (2, 18). In some patients, striatal injury may occur without clinically apparent crisis, this has been termed insidious-onset GA-I (27). Study shows that a few patients with insidious motor delay have suffered striatal injuries before or shortly after birth, followed by latent periods of several months before disability was apparent (28). Principles of emergency treatment in GA-I: (1) a high-energy intake to prevent or reverse a catabolic state; (2) decrease or omitting natural protein for 24 (-48) h to reduce neurotoxic GA and 3-OH-GA; (3) detoxification measures and prevention of secondary depletion carnitine supplementation; (4) balance electrolytes and pH status (29). If the child does not develop alarming symptoms (e.g., alteration in level of consciousness, diarrhea, vomiting, irritability, hypotonia, dystonia), antipyretics such as acetaminophen or ibuprofen should be administered if body temperature rises >38.5°C, maltodextrin solutions or comparable carbohydrate supplementations can be given orally according to guideline recommendation, maintenance treatment could be reintroduced stepwise during 48 (−72) h with assessment of individual's state every 2 h. If alarming symptoms evolve, individuals should immediately start emergency treatment in hospital: (1) intravenous injections of Glucose, it may need to provide 12–15 g/kg/24 h of glucose for 0–1 years, 10–12 and 8–10g/kg/24 h for 1–3 and 3–6 year of age, if persistent hyperglycemia >150–180 mg/dL (>8–10 mmol/L) and/or glucosuria occurs, start insulin and adjust the infusion rate according to serum glucose; (2) Protein intake: Stop natural protein for up to 24 h, and reintroduce and increase stepwise until maintenance treatment is reached within 48–72 h, AAM should be administered according to maintenance therapy; (3) L- carnitine: 100 mg/kg/d *via* intravenous infusion. An example of a successful nutritional intervention was an 11-month male infant, who was diagnosed with GA-1 at 25 days of age and treated with L-carnitine (50 mg/kg/day) immediately. At day 30, the dose was increased to 100 mg/kg/day while starting a low lysine diet (100 mg/kg/day) by breast-feeding, combined with 1.0 g/kg/day of lysine- and tryptophan-free AAMs,. He was admitted due to severe acute subdural hemorrhage after a minor head trauma, Metabolic emergency treatment with IV glucose infusion (15g/kg/d), L-carnitine (200 mg/kg/d) and multiple vitamin supplementations was immediately started. Natural protein and amino acid mixtures were both terminated. Administration of lysine from natural protein and lysine-free and tryptophan-free AAMs by nasogastric tube was initiated within 48 h after surgery. At 14 days. glucose infusion was terminated and L-carnitine dose was 100 mg/kg/day. The patient's oral intake without nasogastric tube recovered to the same level before subdural hemorrhage and the boy was discharged on day 35 (30).

## GA-II

### Pathogenesis of GA-II

**Figure 3:**
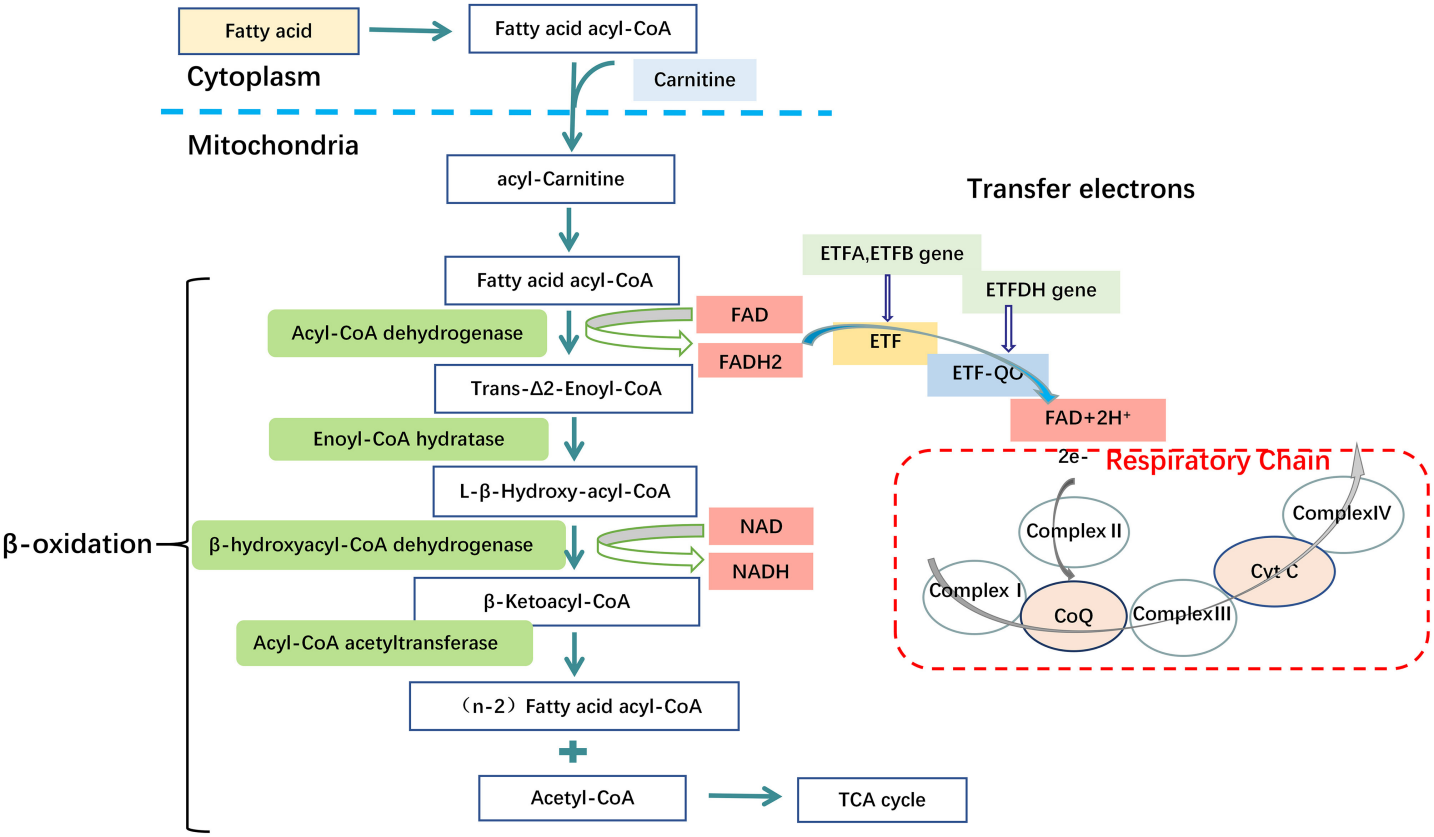
Metabolic disorders of fatty acid in GA-II. Mutations in the Electron Transfer Flavoprotein (ETFA, ETFB) or Electron Transfer Flavoprotein Dehydrogenase (ETFDH) genes. These genes encode the α/β subunits of Electron Transfer Flavoprotein (ETF) and Electron Transfer Flavoprotein-Ubiquinone Oxidoreductase (ETF-QO) respectively. ETF and ETF-QO are the key transporters in the process of electron transfer of fatty acid β-oxidation. In mitochondria, ETF, which is located in the matrix, receives electrons from several FAD-containing acyl-CoA dehydrogenases involved in fatty acid oxidation. ETF transfers electrons to ETF-QO, located in the inner mitochondrial membrane and, subsequently, electrons are passed to ubiquinone in the respiratory chain. Consequently, a dysfunction of ETF or ETF-QO causes the final process of fatty acid β-oxidation to fail, thereby leading to disturbed ATP biosynthesis from fatty acid, excessive lipid accumulation and disturbed gluconeogenesis, Complex I (NADH: ubiquinone reductase), complex II (succinate: ubiquinone reductase), complex III (ubiquinol: cytochrome C oxidoreductase or cytochrome bc1 complex), complex IV (cytochrome c oxidase).

Glutaric acidemia type II (GA-II), also known as Multiple Acyl-Coenzyme A Dehydrogenase Deficiency (MADD), is an autosomal recessive genetic disorder of fatty acid, some amino acid and choline oxidation, caused predominately by mutations in the α/β-subunit of Electron Transfer Flavoprotein (ETF, encoded by ETFA, ETFB) or Electron Transfer Flavoprotein-Ubiquinone Oxidoreductase (ETF-QO, encoded by ETFDH) (31–33) (Figure 3). The loci are on chromosomes 15q23-q25, 19q13.3, and 4q32-qter, respectively (34, 35). GA-II is characterized clinically by hypo- or nonketotic hypo-glycemia and metabolic acidosis, biochemically by accumulation and excretion of substrates (or their derivatives) of the many (at least eight) flavoprotein dehydrogenases which transfer electrons to ETF (36). Defect of ETF or ETF-QO, the protein which transfers electrons from ETF to the ubiquinone pool of the respiratory chain, results in compromised fatty acid oxidation, with consequent impaired adenosine triphosphate (ATP) synthesis, insufficient gluconeogenesis and excessive lipid accumulation in different organs (6). Biochemical investigations can demonstrate elevated serum fatty acylcarnitine of various lengths, and a variety of organic acids in urine. Liver biopsies can indicate fatty metamorphosis, and muscle biopsies a vacuolar myopathy with lipid accumulations (37, 38).

GA-II can be classified into three categories by highly variable clinical feature: A neonatal-onset form with congenital anomalies; most commonly cystic or dysplastic kidneys (type 1), a neonatal-onset form without congenital anomalies (type 2), and a late-onset form (type 3) (5, 39). There are close relationships between ETF/ETFDH genotype and clinical GA-II phenotype. The neonatal-onset forms are usually fatal and typically present with severe nonketotic hypoglycemia, metabolic acidosis during the first days of life, which are relatively more common in ETFA and ETFB pathogenic variants (types 1 and 2) (40). The symptoms and age at presentation of late-onset forms is extremely variable and a majority of individuals has pathogenic variants in ETFDH gene. In adolescents and adults, muscular or cardiac symptoms or episodic vomiting are usually first suggestive features (41, 42), while neonates usually present with severe metabolic decompensations including metabolic acidosis, nonketotic hypoglycemia, hyperammonemia, hypotonia, coma and cardiomyopathy (39, 43). the organic aciduria is often intermittent and only evident during periods of illness or catabolic stress often triggered by infection or fasting stress. The phenotype is also influenced by environmental factors like cellular temperature, especially apparent in milder forms (type 3), it could envisage decreased levels of active mutant ETF enzyme in the case of fever (43).

### Nutrition Therapy

The clinical condition typically deteriorates despite treatment and prognosis of neonatal-onset forms is very poor. Newborns of type 1 become symptomatic within a few hours after birth and will, in most cases, have died within the first week of life. Newborns of type 2 usually present within a few days after birth with metabolic decompensation. Most children do not survive the initial episode. Those who do survive usually die later in infancy either due to hypertrophic cardiomyopathy or recurrence of metabolic decompensation that resembles the Reye syndrome (34, 44, 45). There is significant genetic heterogeneity in GA-II with some genotype-phenotype correlations, specific mutations in ETFDH have been associated with riboflavin-responsive symptoms as well as a myopathic form related to secondary CoQ10 deficiency (35). Nutritional treatment has been somewhat more successfully improved the outcome when applied to patients with later-onset GA-II, including: (1) a low-protein/low-fat/high-carbohydrate diet and avoid fasting (46); (2) supplementation with riboflavin to stabilize the ETF/ETFDH complex; (3) carnitine supplementation to maintain normal carnitine level; (4) coenzyme Q10 supplementation. The treatment strategies in conditions of the GA-II are summarized in Table 2.

**Table 2:** The currently treatment strategies of GA-II.

**GA-II**	**Maintenance treatment**				**Emergency treatment**
Treatment principles	1. Diet: w-fat, low-protein, high-carbohydrate (lipid restricted to 25% of total calories and long-chain fatty acids (LCFA) should be reduced); avoid fasting. 2. Supplementation: L-cartinine:1–4 g/d or 50 mg/kg/d; riboflavin 60–200 mg/d; CoQ10 5–15 mg/d.				1. iv high-dose glucose (8–12 mg/kg/min) to maintain blood glucose >100 mg/dL (start insulin infusion if hyperglycemia) 2. Correct metabolic acidosis, consider hemodialysis or hemofiltration to measure severe hyperammonemia. 3. iv L-carnitine at 50–100 mg/kg/day if severe carnitine deficiency 4. Avoid administration of intravenous intralipids during an acute metabolic crisis.
**Special condition**	**Pregnancy**				
Week	8 weeks	12 weeks	24 weeks	34 weeks	39 (elective cesarean section)
Energy intake	27 (Kcal/kg/d)	29 (Kcal/kg/d)	30 (Kcal/kg/d)	26 (Kcal/kg/d)	delivery and in the immediate postoperative period: 1. iv carnitine 3,000 mg/day 2. Isotonic solution with 10% dextroseat variable rate to maintain normoglycaemia.
Diet	1. A six-meal diet high in carbohydrate (>50% of total calories), very low in fat, protein (10 g/day in the 2 trimester and 30 g/day in 3rd trimester). 2. A glucose supply (25–50 g) every 2 h was recommended in case of discomfort or episodic vomiting.				
L-carnitine	3,000 (mg/kg/d)	3,000 (mg/kg/d)	4,000 (mg/kg/d)	4,000 (mg/kg/d)	
Riboflavin	100 (mg/kg/d)	100 (mg/kg/d)	100 (mg/kg/d)	100 (mg/kg/d)	
Pyridoxine	300 (mg/day)	300 (mg/day)	300 (mg/day)	300 (mg/day)	

#### Maintenance Therapy

##### Dietary Treatment

A low-fat, low-protein, high-carbohydrate diet is beneficial, although the long term treatment of late-onset GA-II patients is still challenging, fat consumption should be restricted to 25% of total calories, and the amount of long-chain fatty acids (LCFA) should be reduced (46). The main caution is the avoidance of fasting, by not depending on β-oxidation for energy, the accumulation of toxic intermediate metabolites is avoided and the development of the most critical symptoms is minimized. In daily life, it may be difficult to implement a diet plan that limits fat and protein strictly. Diet such as limitation of protein should be started in consultation with a metabolic dietitian and should be combined with medical formulas to ensure adequate metabolic control and appropriate growth in infants, children and adolescents (39).

##### High-Dose Riboflavin Supplementation

**Figure 4:**
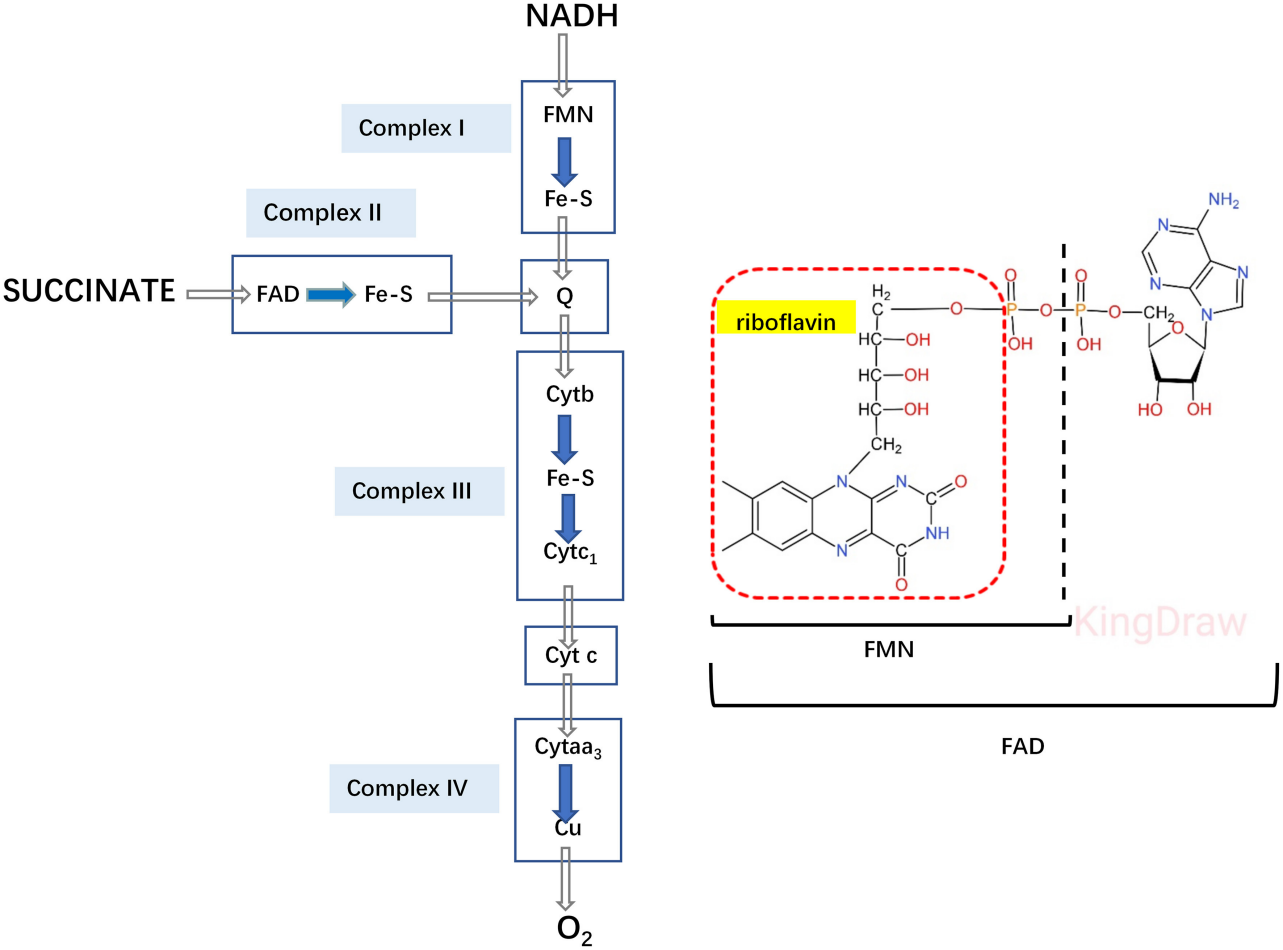
Electron transfer system in the respiratory chain. Riboflavin is converted to FAD, as cofactor for both ETF and ETFDH, also enter as a coenzyme for complex I and II in the respiratory chain.

Riboflavin supplementation should be tried in all GA-II patients irrespective of the molecular genetic cause 98% of late-onset GA-II respond to riboflavin (43). Riboflavin is precursor of flavin adenine dinucleotide (FAD), which is a cofactor for ETF, ETFDH and several mitochondrial enzymes, such as the Acyl-CoA Dehydrogenases, but also enter as coenzyme in complexIand complex II of respiratory chain (47, 48) (Figure 4). Riboflavin deficiency may be due to different mechanism: decreased cellular riboflavin uptake and decreased FAD synthesis; decreased FAD transport into mitochondria; abnormal binding of FAD to apoenzymes; increased catabolism for increased FADPase (49). The therapeutic efficacy of riboflavin replacement for GA-II was first reported by Gregersen in 1982 (50). By increasing the intra-mitochondrial FAD level, high dose riboflavin (100–300 mg/day) supplementation in late-onset forms promote FAD binding to ETF or ETF-QO, and thereby stabilizing the ETFDH enzyme and enhances its activity (51). Riboflavin is commonly considered the most important therapeutic agent for GA-II, Besides its well-known effects on motor function and severe hypotonia, a previous study observed beneficial improvement on intellectual disability, although it was difficult to differentiate whether the effects were due to riboflavin supplementation alone or the combination of multiple drugs (52). The study showed Riboflavin-responsive GA-II to be associated with defects in the ETFDH gene (35, 51).

##### L-carnitine Supplementation

**Figure 5:**
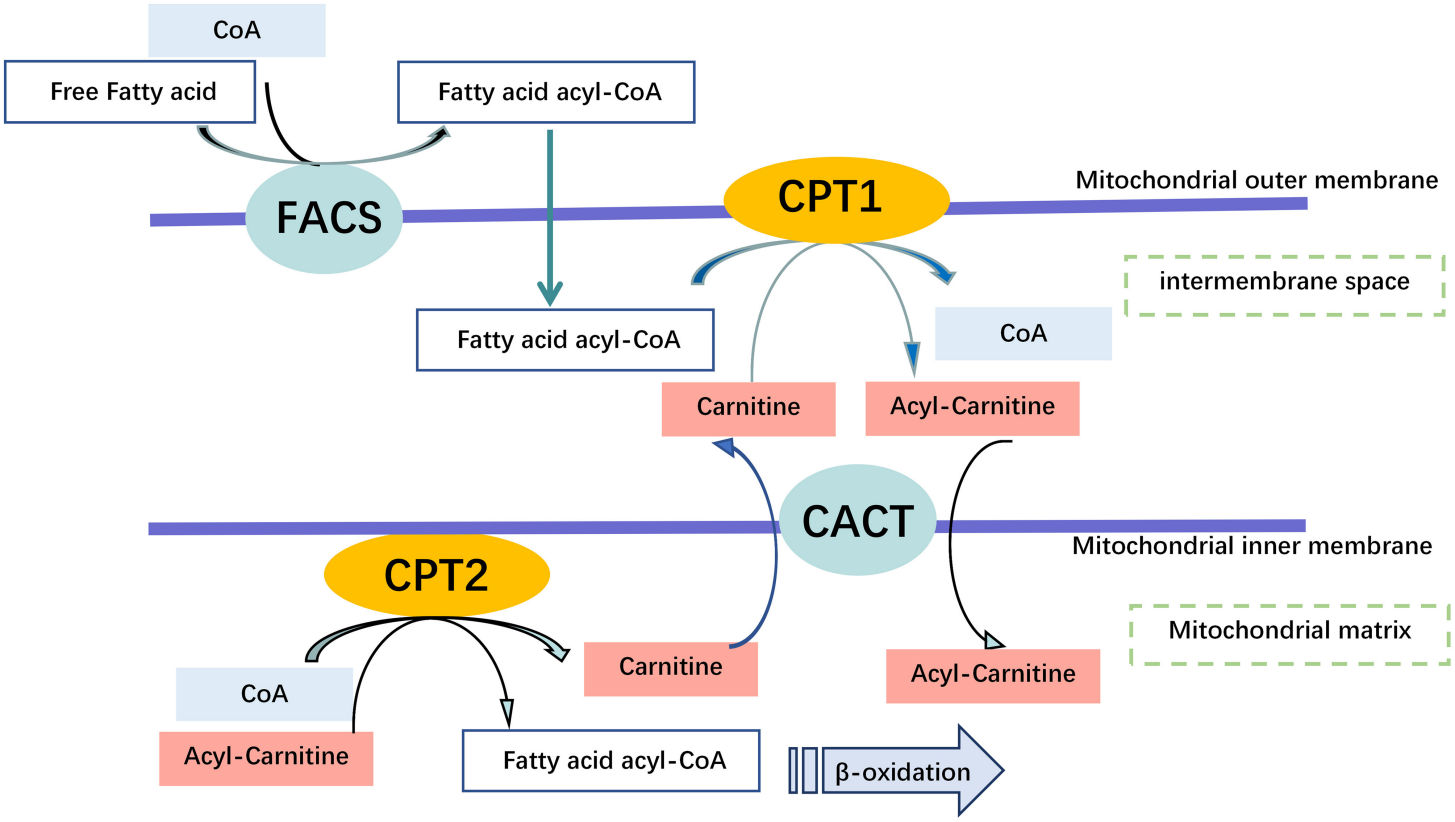
Carnitine Shuttling System. Once the free fatty acid reaches the Mitochondrium, it is processed into a fatty acid acyl CoA molecule by Fatty Acyl-Coenzyme A Synthetase (FACS) to cross the outer mitochondrial membrane. Carnitine is used by Carnitine Palmitoyl transferase 1 (CPT1) to synthesize acyl carnitine, which is shuttled across the inner mitochondrial membrane by Carnitine Translocase (CACT). Finally, acyl carnitine is converted back into carnitine, and a fatty acid acyl-CoA molecule by carnitine palmitoyl transferase 2 (CPT2) for β-oxidation within the mitochondrial matrix, and transport of free carnitine back to the cytoplasm by CACT.

Carnitine is involved in the transport of long-chain fatty acids from the cytoplasm to the mitochondrial matrix for β-oxidation, defects of the carnitine transporter result in the accumulation of long-chain fatty acids and triglycerides (Figure 5). Due to the loss of carnitine conjugates *via* the urine, GA-II patients are prone to carnitine deficiency, which may require oral carnitine (L-carnitine 50–100 mg/kg/day) supplementation (39, 53).

##### Coenzyme Q10 Supplementation

Coenzyme Q (CoQ), also known as ubiquinone, is localized in the inner mitochondrial membrane, where it acts as a mobile electron and proton transporter from electron transport chain complex I and complex II to complex III (Figure 3). CoQ also links mitochondrial fatty acid β-oxidation to the electron transport chain by accepting electrons from ETF-QO and passing them on to complex III (54, 55). A recent study showed that the late-onset form of GA-II and the myopathic form of CoQ10 deficiency are allelic diseases. Patients who carried autosomal recessive mutations in ETFDH exhibited a secondary CoQ10 deficiency and decreased activities of respiratory chain complexes in skeletal muscle biopsies (56). CoQ10 treatment can compensate for an increased mitochondrial oxidative stress in fibroblasts from riboflavin-responsive-GA-II patients, which is most likely caused by misfolded variant ETF-QO proteins with decreased CoQ10 binding (57). Like riboflavin, CoQ10 supplementation (60–240 mg/day) should be tried in all late-onset GA-II, especially on the protracted course (39). For example, a 16-year-old male patient was hospitalized due to muscle weakness. Genetic test results confirmed GA-2 for ETFDH deficiency. CoQ10 treatment was administered: first 40 mg for 3 months, then dose was adjusted to 20 mg for 6 months, followed by the change of 10 mg for long-term use. The patient's condition significantly improved after 3 months. At the 8th year follow-up, his blood CK was normal, a muscle biopsy revealed no muscle vacuolar fibers and increase in lipid droplets. Symptoms were significantly alle*via*ted after appropriate treatments with CoQ10 (58).

#### Emergency Therapy

Acute symptoms, such as lethargy, encephalopathy, intractable vomiting, rhabdomyolysis or progressive coma often occur in the setting of intercurrent illness and/or inadequate caloric intake due to poor appetite or prolonged fasting or following vaccination. Intercurrent infections, poor oral intake, vomiting, or diarrhea can precipitate metabolic decompensation and lead to vomiting, lethargy, metabolic acidosis, lactic acidosis, and coma. Treatment should be started by intravenous fluid with high-dose glucose (8–12 mg/kg/min) to maintain blood glucose >100 mg/dL. Furthermore, if hyperglycemia is diagnosed insulin has to be given and severe metabolic acidosis has to be countered by sodium bicarbonate (pH <7.10 or bicarbonate <10 mEq/L). Hemodialysis or hemofiltration can be considered to treat severe hyperammonemia and intravenous levocarnitine treatment at 50–100 mg/kg/day has to be started if severe carnitine deficiency is diagnosed. Finally, administration of intravenous intralipids should be avoided during an acute metabolic crisis (39).

#### Specific Physiological Conditions

For most of the inherited diseases there is a lack of specific guidelines for the management during specific physiological conditions such as pregnancy; therefore, experience from isolated case reports is particularly valuable.

##### Pregnancy

It has been reported that several young woman with GA-II have given birth to a healthy child without any complication (6, 59, 60). In order to meet the increased protein demand during pregnancy, it may need a six-meal diet high in carbohydrate (>50% of total calories), very low in fat, but supplemented with protein (10 g/day in the 2th trimester and 30 g/day in 3rd trimester) to meet the increased protein demand. In addition, a glucose supply (25–50g) every 2 h was recommended in case of discomfort or episodic vomiting, along with oral carnitine (3,000 mg/day and increase to 4,000 mg/day because of an increased sense of fatigue), riboflavin (100 mg/day) and pyridoxine (300 mg/day), the clinical and biochemical profile remained substantially stable during pregnancy demonstrates careful clinical monitoring associated with an adequate nutritional and medical management may improve pregnancy outcome in women (6).

##### Treatment of Critically Ill Infants

As soon as the diagnosis of GA-II is suspected, treatment with riboflavin and carnitine must be started, together with a low protein/fat, high carbohydrates diet. Treatment with insulin has been reported can improve the metabolic condition of critically ill infants with GA-II, which considered to improve glucose utilization, after subcutaneous injection of one unit insulin every 4 h, this 10 months old infants hypoglycemia corrected and glucosuria disappeared. A glucose enriched diet resulted in an dramatically increase in muscular strength and weight gain. After insulin therapy gradually withdrawing, his mental and motor development were progressing, though a slight hypotonia remained (61).

## Conclusion

Glutaric acidemia (GA) represent a group of genetic disorders characterized by excessive and pathological organic acid accumulation in urine and intermediates accumulation in blood and other organs. The underlying causes of disease have been linked to genes associated with certain amino acid and fatty acid metabolism. Based on the irreversible damage of encephalopathic crises and poor prognosis of acute metabolic crisis for GA-I and GA-II, neonatal screening, early and correct diagnosis is crucial. In those regions where newborn screening is not performed, a high index of clinical suspicion is necessary. Current treatment strategies of GA are based on nutrition therapy, including dietary treatment combined with other supplements, such as L-carnitine, riboflavin or CoQ10. Furthermore, strict emergency treatment is administered during putatively threatening episodes, which could prevent the onset of striatal injury and irreversible neurological symptoms in the majority of neonatally diagnosed patients. There is clear progress in improving the quality of life and reducing mortality in this patient population. However, GA is difficult to study by currently accepted standards of medical evidence. Small patient numbers, substantial regional differences in screening and medical care limit the large placebo-controlled trials and impeded general consensus that most treatments are based on according to individual cases or expert opinions. More trials are needed to define efficacious treatments and avoid unnecessary treatments. For instance, L-carnitine, vitamin, and CoQ10 supplements may be overused and new therapeutic approaches are currently in clinical trials that may improve therapeutic options in the near future.

## Author Contributions

XW: conceptualization, methodology, and funding acquisition. QL: writing–original draft. HK: writing–review and editing. YH: investigation, resources, data curation, and revised the manuscript. CY, LF, YH, YZ, YS, HL, and HM: investigation, resources, and data curation. All authors contributed to the article and approved the submitted version.

## Funding

This work was supported by the National Natural Science Foundation of China (82104185), the Anhui Provincial Natural Science Foundation (2008085QH400), the open fund of the Key Laboratory of Anti-inflammatory and Immune Medicine, Ministry of Education, P.R. China (Anhui Medical University, KFJJ-2020-03), and the Clinical Science Foundation of Anhui Medical University (2021xkj148).

## Conflict of Interest

The authors declare that the research was conducted in the absence of any commercial or financial relationships that could be construed as a potential conflict of interest.

## Publisher's Note

All claims expressed in this article are solely those of the authors and do not necessarily represent those of their affiliated organizations, or those of the publisher, the editors and the reviewers. Any product that may be evaluated in this article, or claim that may be made by its manufacturer, is not guaranteed or endorsed by the publisher.
